# Frog Fibres: What Muscle Architecture Can Tell Us About Anuran Locomotor Function

**DOI:** 10.1002/jmor.70016

**Published:** 2024-12-17

**Authors:** Alice Leavey, Christopher T. Richards, Laura B. Porro

**Affiliations:** ^1^ Centre for Integrative Anatomy, Cell and Developmental Biology University College London, Bloomsbury London UK; ^2^ Structure and Motion Laboratory Royal Veterinary College—Camden Campus, Comparative Biomedical Sciences London UK

**Keywords:** diceCT, fibre tracking, GoodFibes, hindlimb, locomotion

## Abstract

Muscle fibre architecture is an important aspect of anatomy to consider when estimating muscle properties. How fibre architecture varies across species specialising in different locomotor functions is not well understood in anurans, due to difficulties associated with fibre extraction in small animals using traditional methods. This paper presents the first digital analysis of fibre architecture in frogs using an automated fibre‐tracking algorithm and contrast‐enhanced µCT scans. We find differences in hindlimb muscle fibre architecture between frogs specialising in different locomotor modes, as well as examples of many‐to‐one mapping of form to function. The trade‐off between fibre length and muscle physiological cross‐sectional area, and therefore contractile speed, range of motion and muscle force output, differs significantly between jumpers and swimmers, but not walker‐hoppers. Where species place on this functional spectrum of fibre architecture largely depends on the muscle being examined. There is also some evidence that fibre length may be adjusted to increase contractile speed without undertaking the metabolically expensive process of growing and maintaining larger muscles. Finally, we make a detailed outline of the remaining gaps in our understanding of anuran fibre architecture that can now be addressed with this valuable digital method in future research.

## Graphical abstract

Our study utilises a new, automated approach to digitally extracting muscle fibres from μCT data, allowing us to present the first digital analysis of muscle fibre architecture in frogs, including some of the world's smallest vertebrates. We find that the trade‐off between fibre length and physiological cross‐sectional area, and therefore contractile speed, range of motion and muscle force output, differs significantly between frogs specialising in jumping and swimming, but not between these groups and frogs specialising in walking/hopping.

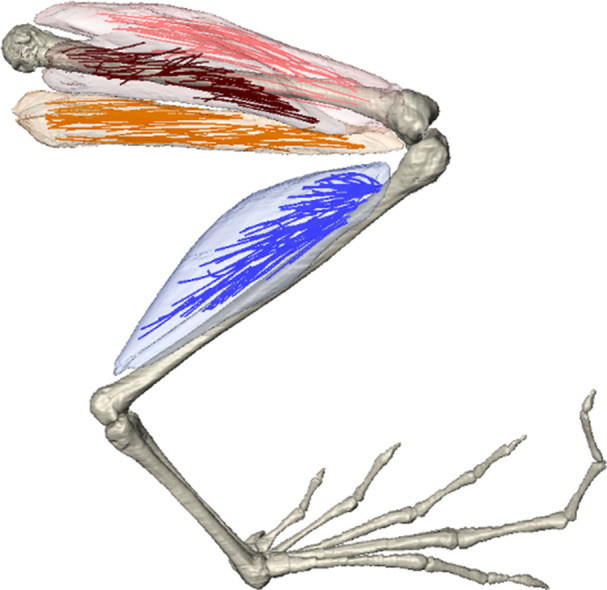

## 1 Introduction

Comparative anatomists have long studied the diversity of muscle structure to help explain the great variety in mechanical function seen across vertebrates. Aside from physiological differences in contractile properties of individual fibres, the architectural arrangement of the fibres can tremendously impact the mechanical function of whole muscles (Lieber and Bodine‐Fowler 1993). Specifically, muscle fibres can be arranged in complex ways, differing in how they are angled relative to the force‐producing axis (i.e., degree of pennation) and rarely stretching along the entire length of the muscle from origin to insertion, even within parallel‐fibred muscles (Rabey et al. 2015; Perry and Prufrock 2018). For a given volume, muscles with parallel fibres have higher maximum excursions, creating larger functional ranges of motion, and can produce faster contractile velocities (Lieber and Fridén 2000). Muscles of the same volume with a higher pennation angle tend to have shorter, more tightly packed fibres which can produce higher forces (Gans 1982; Sacks and Roy 1982; Powell et al. 1984). Muscles cannot be optimised for both contractile velocity and maximum force generation without incurring detrimental functional trade‐offs (Rabey et al. 2015). Muscle architecture is therefore likely under strong selection as it has important implications for ecologically relevant performance traits.

Frogs are frequently used as model organisms for comparative analyses of functional morphology across evolutionary time. Anatomical changes in their largely conserved body plan can enable anurans to employ many different locomotor functions, which in turn grants them access to various ecological niches (Jorgensen and Reilly 2013; Enriquez‐Urzelai et al. 2015; Nauwelaerts, Ramsay, and Aerts 2007; Gomes et al. 2009; Moen, Irschick, and Wiens 2013; Tulli et al. 2016; Citadini et al. 2018; Moen 2019; Leavey, Richards, and Porro 2023, 2024). Previous studies have found that the masses of several key hindlimb muscles are important predictors of locomotor performance in frogs (Enriquez‐Urzelai et al. 2015; Nauwelaerts, Ramsay, and Aerts 2007; Calow and Alexander 1973; Emerson 1978; Choi and Park 1996; Gillis and Biewener 2000; Astley 2016). However, there were several instances recently highlighted in a large comparative study of hindlimb muscles across all the primary locomotor functions where the mass and length of many muscles does not differ significantly between locomotor modes as expected, implying that there is more influencing motion than gross muscle size (Leavey, Richards, and Porro 2024). Physiological cross‐sectional area (PCSA) is likely more representative of muscle function compared to muscle mass, as it incorporates pennation angle and fibre length into estimates of a muscle's force‐producing capacity (Rabey et al. 2015; Sacks and Roy 1982; Powell et al. 1984; Biewener 1989). However, differences in muscle architecture in frogs have usually only been examined in relation to jumping performance, and only within one muscle across a small number of species (Azizi and Roberts 2014; Mendoza and Azizi 2021), or between many muscles within the same species (Calow and Alexander 1973; Lieber and Brown 1992; Kargo and Rome 2002). Even then, few studies examine isolated fibre lengths (e.g., after nitric acid digestion) (Astley 2016; Lieber and Brown 1992; Kargo and Rome 2002). Astley (2016) is the most comprehensive study so far, having analysed two muscles across 14 species in relation to jumping, swimming and walking. However, all of the aforementioned studies measured less than 25 fibres per muscle, which can have significant consequences for estimates of muscle function (Charles et al. 2022). The general lack of phylogenetic coverage and consideration for locomotor functions besides jumping prevents comprehensive evaluations of the role of each muscle during motion.

Much of the reason behind this scarcity of frog muscle architecture studies is because individual muscle fibres are notoriously difficult to isolate intact using physical dissection, especially in particularly small animals and/or muscles (Lieber and Fridén 2000). In the last decade, significant progress has been made in attaining sufficient contrast‐enhanced µCT scan resolution to examine minute internal structures, including muscle fibre arrangement (Gignac and Kley 2014; Nyakatura et al. 2019; Dickinson et al. 2020). Furthermore, methods for automated fibre recognition and tracking have been recently developed in ImageXd (Nyakatura et al. 2019; Kupczik et al. 2015; Dickinson, Stark, and Kupczik 2018), Amira/Avizo (Sullivan et al. 2019; Peeters et al. 2020; Holliday et al. 2022) and Python (Püffel et al. 2021) (see Katzke et al. 2022 for a review). These techniques have never been applied to frogs, presenting a unique opportunity to study the complex relationship between anatomy and function using a relatively high number of fibres, muscles and species.

**Figure 1:**
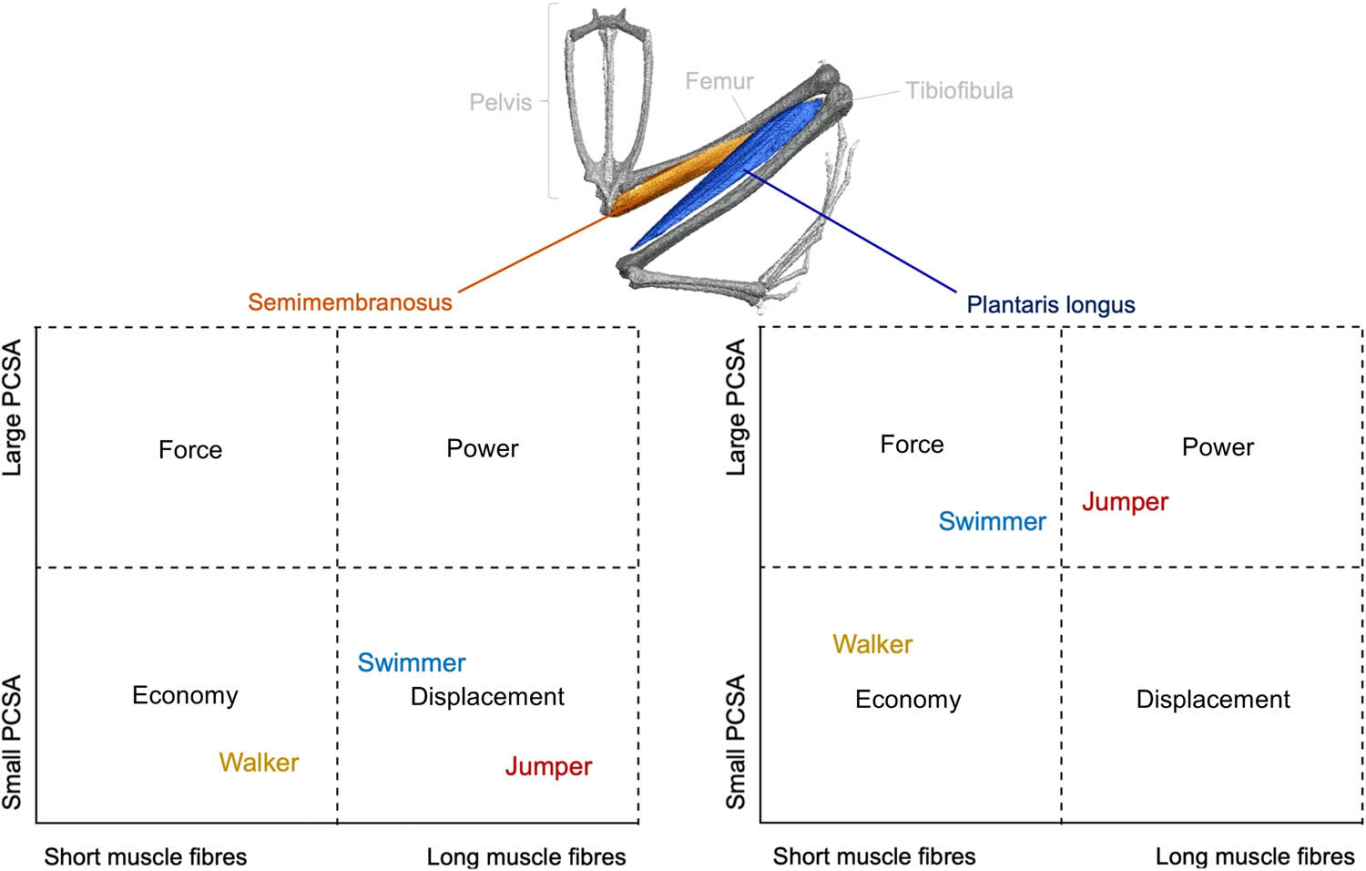
Functional space plots hypothesising how the trade‐off between fibre length and physiological cross‐sectional area (PCSA) can differ between both locomotor modes (H1) and muscles specialising in different functions (H2), using a parallel‐fibred hip extensor (semimembranosus) and pennate ankle extensor (plantaris longus) as examples (dorsal view). The product of these axes is the volume of the muscle, where the largest muscles are in the power quadrant, while the smallest muscles are in the economy quadrant. The anatomical model shown is of *Leptodactylus poecilochilus* (voucher number: CAMZN R.16735.A) and was created in Amira (Version 2020.2).

Given these knowledge gaps and recent advancements in fibre tracking technology, the overarching aim of this paper is to determine the link between hindlimb muscle fibre architecture and locomotor mode, specifically for frogs that specialise in jumping, swimming or walking/hopping. Since each muscle plays a different functional role in locomotion, we also aim to investigate whether differences in fibre architecture between locomotor modes depend on the muscle type (pennate vs. parallel‐fibred). In previous literature, functional space plots of PCSA against fibre length have frequently been used as intuitive ways of identifying areas of muscle specialisation and comparing potential trade‐offs between species (see Martin et al. 2020 for a summary). Here, Figure 1 describes how muscle function can be inferred from fibre architecture, and explains the hypotheses addressed in this paper. This type of plot separates out muscles (and, in this case, species with different locomotor modes) that are likely specialised for different functions— producing high forces, high contractile velocities and large ranges of motion, or high power (force times velocity) (Martin et al. 2020; Böhmer et al. 2018). The final category, ‘economy’, describes muscles with specialisation for low energy expenditure (Figure 1). For instance, since frogs that primarily walk/hop have smaller, less muscular legs than frogs which specialise in jumping and swimming (Leavey, Richards, and Porro 2024; Astley 2016), walker‐hoppers are expected to occupy this economy section of the functional space plot (Figure 1). Additionally, where each species places on the functional plot may depend on the muscle being examined. For example, the size of the plantaris longus, an ankle extensor, is positively associated with both swimming and jumping performance via its role in power production (Enriquez‐Urzelai et al. 2015; Gillis and Biewener 2000; James and Wilson 2008), while the semimembranosus, a parallel‐fibred hip extensor (Kargo and Rome 2002), has faster contractile properties in jumpers than in swimmers (Figure 1; Astley 2016). Therefore, we hypothesise that: (H1) the trade‐off between PCSA (muscle force) and fibre length (muscle contractile speed, range of movement) for each muscle will differ between locomotor modes; and (H2) differences in fibre architecture between locomotor modes will depend on the hindlimb muscle being examined.

## 2 Methods

### 2.1 Data Selection

Out of the 30 species used in Leavey, Richards, and Porro (2024), who performed digital dissections of the hindlimb musculoskeletal anatomy, 10 have a µCT scan with a suitable resolution for visualising individual fibres— *Arthroleptis tanneri* (terrestrial jumper; TJ), *Ischnocnema guentheri* (TJ), *Leptodactylus poecilochilus* (TJ), *Rana temporaria* (TJ), *Sechellophryne gardineri* (TJ), *Barbourula busuangensis* (swimmer; AQ), *Occidozyga laevis* (AQ), *Telmatobius brevipes* (AQ), *Eupsophus roseus* (walker‐hopper; WH), and *Paedophryne verrucosa* (WH). Walker‐hoppers are defined as frogs which choose to perform asymmetrical walking gaits more often than they jump and hop, and they cannot perform a leap greater than eight times their snout‐vent length (SVL) (Emerson 1979; Reilly et al. 2015). Information on locomotor mode was gathered from the literature (e.g., Jorgensen and Reilly 2013) and through exchanges with researchers who have conducted first‐hand behavioural observations in the field (Dr. Andrew Gray and Dr. Dave Blackburn, Personal Communications). A review of the literature indicated which hindlimb muscles were the most important determinants of locomotor performance, resulting in a priority list of muscles to test (Enriquez‐Urzelai et al. 2015; Nauwelaerts, Ramsay, and Aerts 2007; Calow and Alexander 1973; Gillis and Biewener 2000; Astley 2016; Azizi and Roberts 2014; Mendoza and Azizi 2021; Lieber and Brown 1992; Kargo and Rome 2002). Four muscles from a range of functional groups that most consistently showed high visual resolution were chosen for our study—the gluteus magnus (parallel‐fibred knee extensor), semimembranosus (parallel‐fibred hip extensor), cruralis (pennate knee extensor) and plantaris longus (pennate ankle extensor).

### 2.2 Exporting and Preparing Muscle Image Stacks

The muscles of interest had been digitally dissected for one individual per species by Leavey, Richards, and Porro (2024) in either Amira (Version 2020.2) or VGStudio Max (Version 3.4). To prepare an image stack of each muscle for analysis, muscles were first aligned with the global *Z* axis so that a cross‐section through the fibres could be visualised. In VGStudio Max, the image stack can be exported directly, while scans segmented in Amira require use of the arithmetic module to first isolate the muscle from the rest of the scan using the formula A × (B > 0) (where A is the original image stack and B is the label field of the muscle of interest). To prevent the tracking algorithm passing between neighbouring fibres, the number of grayscale values was minimised by using the ‘unsharp mask’ filter in ImageJ (Supporting Information S2: Figure S1) before fibre tracking.

### 2.3 Automated Fibre Tracking

**Figure 2:**
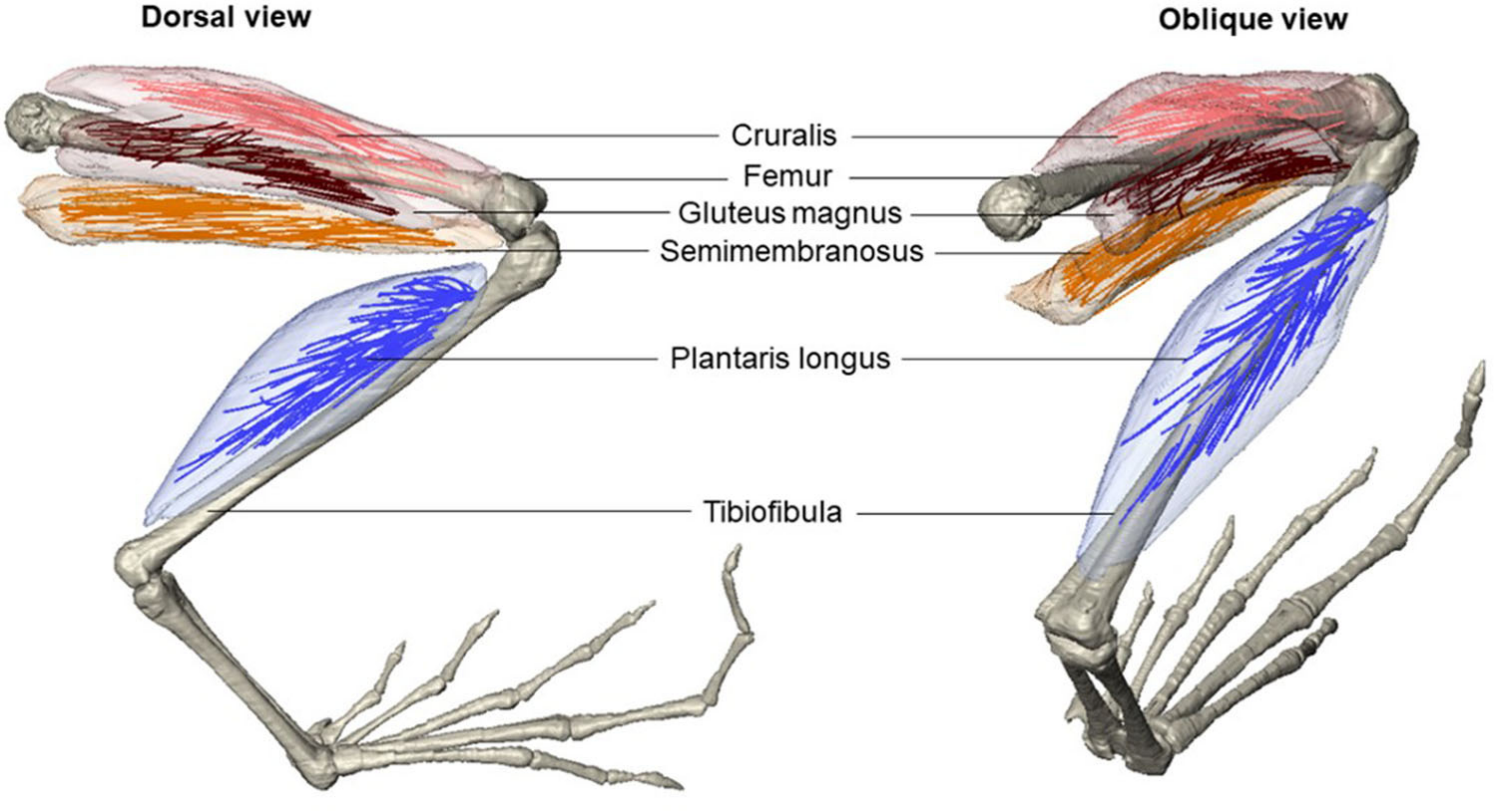
3D visualisation of *Arthroleptis tanneri* (voucher number CAS:HERP:168823) fibres from the four muscles used for analysis, extracted in R (*GoodFibes* package) and visualised in Amira (Version 2020.2).

Fibre extractions and measurements were carried out in R (Version 4.3.1) using the *GoodFibes* package (Arbour 2023). First, histogram equalisation (‘equalise.stack’ function) was used to remove all intermediate grayscale values as the automated fibre tracking algorithm works by not passing through black spaces. The appropriate grayscale cut‐off was determined on a case‐by‐case basis for each specimen, where fibres needed to appear isolated from each other as much as possible without disappearing too early (Supporting Information S2: Figure S1). Fibres were tracked using the 'good.fibes’ function— 50 starting points known as ‘seeds’ were used, with each set of seeds starting from five equally distanced scan slices across the muscle length. The algorithm then traces fibres backwards and forwards from each seed until the fibre disappears (i.e., 95% of the surrounding voxels are black). A ‘bound buffer’ of three was used to prevent fibres from running along the edge of the muscle where the iodine is often more concentrated (Arbour 2023). The ‘quality. check’ function was then used to remove any low‐quality fibres (i.e., fibres with high grayscale variation compared to fibre length, and fibres below one‐tenth of the muscle belly length, as this has never been reported in traditional dissections; Astley 2016). The resulting reconstructed muscle fibres were then exported and visualised as STL meshes (Figure 2). Mean fibre length was calculated from the output of the ‘fibre.lengths’ function. The number of high‐quality fibres extracted from each muscle ranged from 40 to 168 (Supporting Information S1: Dataset), which meets the sample size requirement for statistical analysis of mean fibre length (i.e., > 25 fibres; Charles et al. 2022).

### 2.4 Pennation Angle

As pennation angle cannot yet be reliably extracted using the *GoodFibes* package (Arbour 2023), pennation angle of the traced fibres was measured manually within Amira/VGStudio Max. First, the 2D scan plane was aligned with the force‐generating axis (i.e., along the length of the muscle belly) and positioned in the centre of the muscle (Supporting Information S2: Figure S2). Five measurements of the fibre angle relative to the tendon were taken and averaged to improve accuracy. In line with previous studies, pennation angle was assumed to be constant in all positions and across different layers of muscle (i.e., deep vs. superficial; Kargo and Rome 2002; see Section 4.5).

### 2.5 Statistical Analyses

All of the raw data from our study can be found in the Supporting Information S1: Dataset. Muscle belly volume (MBV) divided by fibre length (FL) calculates the PCSA as a measure of a parallel‐fibred muscle's force‐producing capacity. For a pennate muscle, PCSA is calculated through incorporation of the cosine of the average pennation angle (*f*; Sacks and Roy 1982):

(1) $$\mathrm{PCSA}=\frac{\mathrm{MBV}∙\mathrm{cosf}}{\mathrm{FL}}.$$

To correct for differences in body size, the standard measure for vertebrate skeletal muscle density (1.056 g/cm^3^; Mendez and Keys 1960) was multiplied by the volume of the frog in the μCT scan (extracted from Dragonfly 3D World) to estimate total body mass (real body mass data are only available for four frogs in this data set; Leavey, Richards, and Porro 2024). Since many of our variables are highly correlated with body size (Supporting Information S2: Table S1), log‐transformed PCSA and muscle belly mass were regressed against the log‐transformed estimation of total body mass. The same approach was used for correcting muscle belly length and mean fibre length using SVL (Allen et al. 2010). The resulting residuals were used for subsequent statistical tests, all of which were performed in R (Version 4.3.1). All but three variables of interest were normally distributed (Supporting Information S2: Table S2), and all but one variable showed that ‘white noise’ was the best evolutionary model (see Supporting Information). However, a small sample size means that the phylogenetic signal cannot be accurately estimated (Astley 2016; Münkemüller et al. 2012). Therefore, using the tree from Portik, Streicher, and Wiens (2023) (Supporting Information S2: Figure S3), phylogenetic versions of all analyses were carried out using a Brownian motion model of evolution (see Supporting Information).

To address each of our hypotheses, ANOVA/Kruskal–Wallis and their post hoc tests were used to compare size‐corrected fibre length and size‐corrected PCSA across locomotor modes (addressing Hypothesis 1) and hindlimb muscles (addressing Hypothesis 2). The grayscale cut‐offs used in the fibre tracking algorithm were incorporated as another potential explanatory factor into the comparative models addressing these hypotheses. This was to ensure that any lower cut‐offs required for the algorithm to run did not result in bias leading to abnormally longer fibres in some muscles/species. As sample size is small, corrected Akaike Information Criterion (AICc) was used to determine which models best fit the data. To better address H2, an additional ANOVA was run to account for the potential bias introduced into analyses of PCSA differences between muscles, in that both pennate muscles are frequently much larger than the parallel‐fibred muscles. This test used a version of PCSA where muscle volumes were all made equal to 1 mm^3^.

## 3 Results

### 3.1 Comparisons of Fibre Length

**Figure 3:**
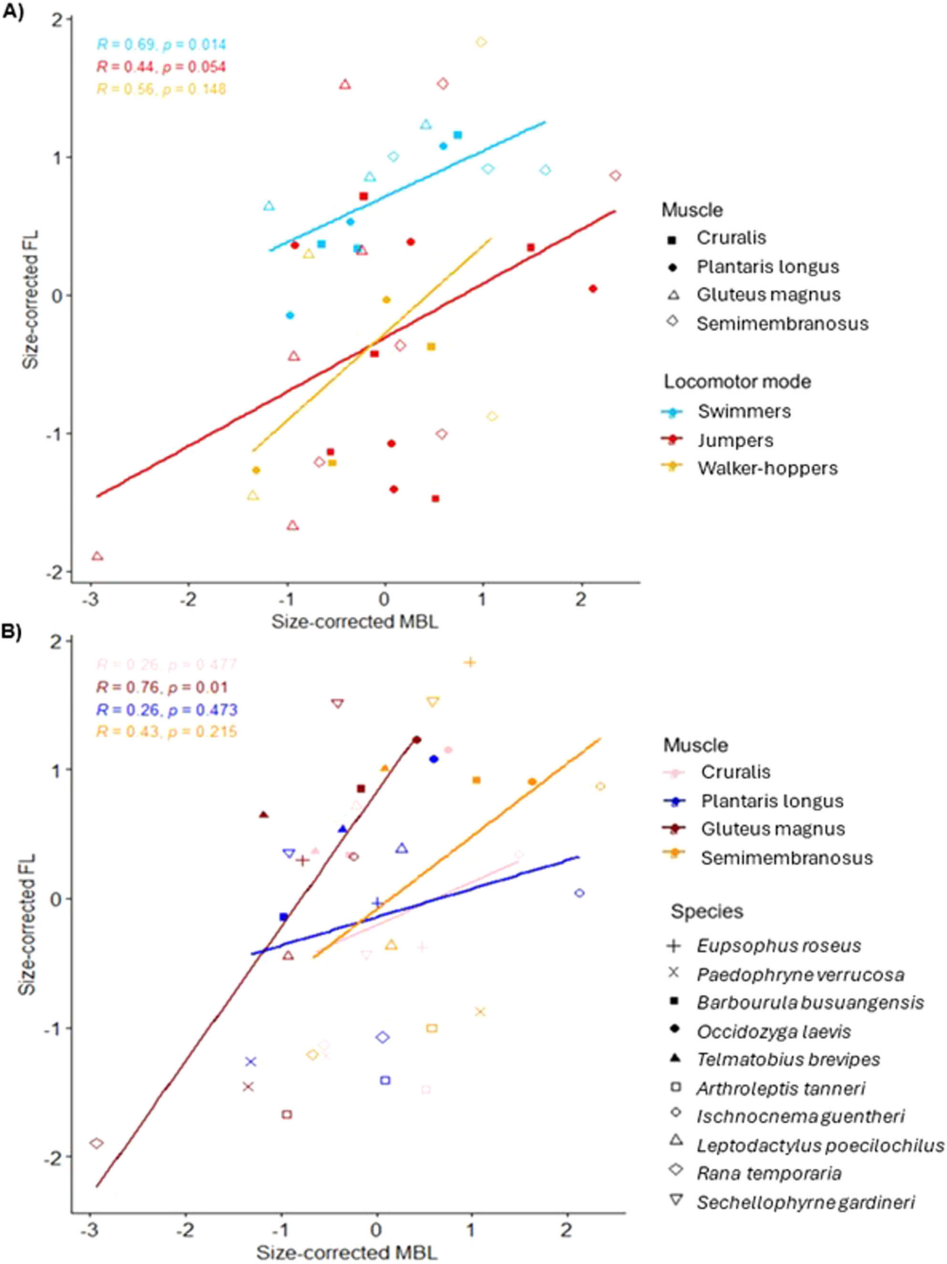
The relationship between size‐corrected fibre length and muscle belly length. There are four points per species; one for each muscle. The data are the same across the two plots, with points colour coded according to either (A) locomotor mode or (B) muscle type. In (A), filled shapes represent pennate muscles and empty shapes represent parallel‐fibred muscles. In (B), empty shapes represent jumpers, filled shapes represent swimmers, and cross‐type shapes represent walker‐hoppers. The statistics reported refer to Pearson's correlation tests for all except the cruralis in (B) which is the result of a Spearman's rank test.

Generally, fibre length increases significantly with the length of the muscle (Pearson's correlation = 0.44, *p* = 0.004). When the data are divided into the different locomotor modes, only swimmers show a significant relationship between fibre length and muscle length, though the general trends are still positive for jumpers and walker‐hoppers (Figure 3). In addition, swimmers show a considerably smaller range of fibre lengths across muscles compared to the other locomotor modes (Figure 3A). When grouped by the specific muscles, fibre length increases significantly with the length of the gluteus magnus (Figure 3B). The other muscles all show a general, nonsignificant increase. The best model of fibre length uses only locomotor mode as the explanatory factor (AICc: 112.15; ANOVA: *F*_(2)_ = 5.76, *p* = 0.007; Supporting Information S2: Table S2). Both jumpers (Tukey HSD: 1.041, *p* = 0.009) and walker‐hoppers (Tukey HSD: 1.127, *p* = 0.027) have significantly shorter fibres than swimmers. In the phylogenetic version of this analysis, there are no significant differences between locomotor modes in fibre length (see Supporting Information).

### 3.2 Comparisons of PCSA

**Figure 4:**
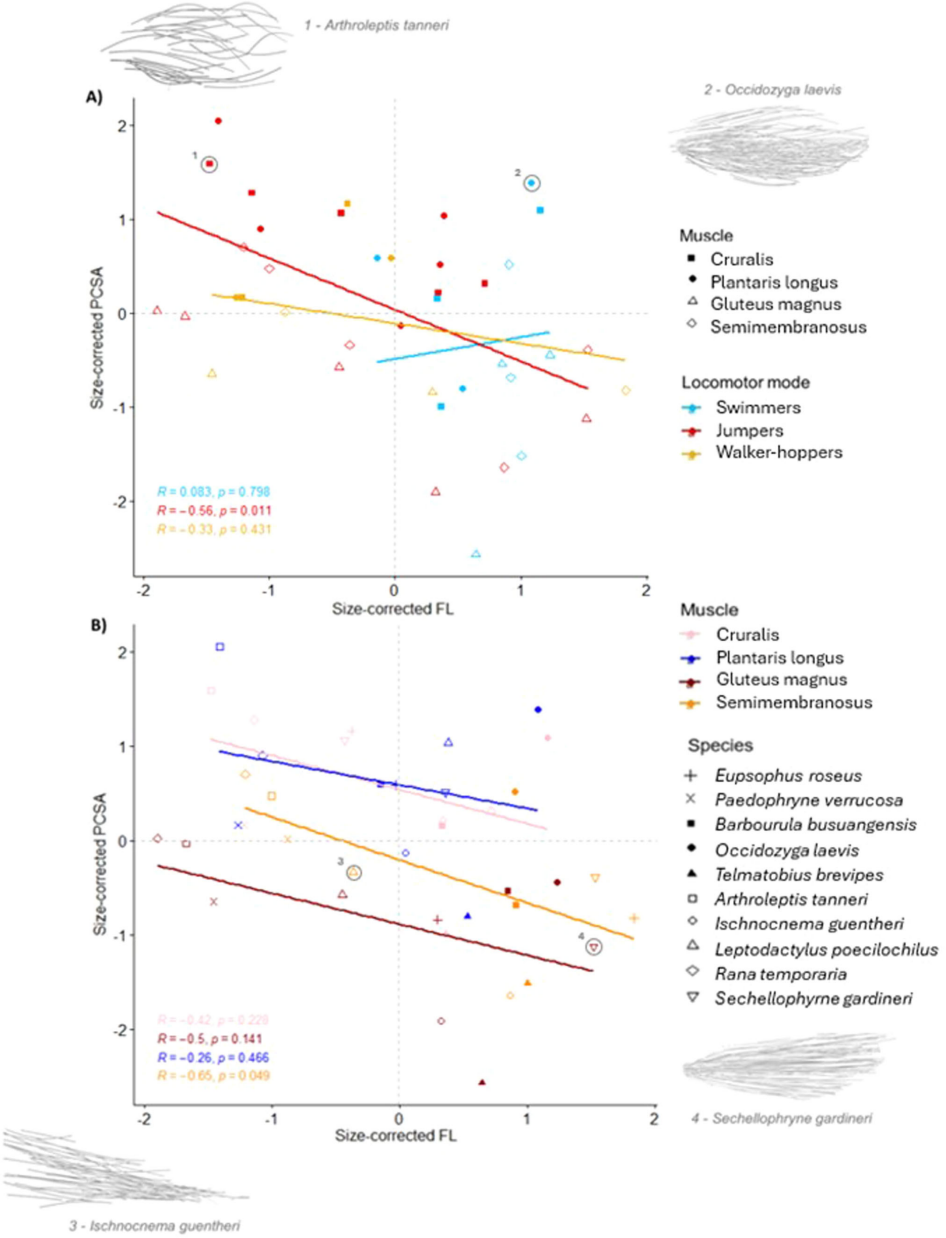
The relationship between size‐corrected physiological cross‐sectional area (PCSA) and fibre length (FL). There are four points per species; one for each muscle. Data are the same across the two plots, with points colour‐coded according to either (A) locomotor mode or (B) muscle type. In (A), filled shapes represent pennate muscles, and empty shapes represent parallel‐fibred muscles. In (B), empty shapes represent jumpers, filled shapes represent swimmers, and cross‐type shapes represent walker‐hoppers. PCSA for the parallel‐fibred muscles simply represents the anatomical CSA (i.e., pennation angle = 0). The grey dashed lines represent the means across each axis, which divide the plot into the four areas of functional space. Each area has an encircled example depicted by the corresponding fibre silhouette. The statistics reported refer to Pearson's correlation tests for all except the semimembranosus in (B) which is the result of a Spearman's rank test.

As expected, PCSA is significantly higher when fibre length is shorter (Pearson's correlation = 0.42, *p* **=** 0.008), indicating a trade‐off between muscle force and contractile speed that is consistent with Equation (1) and the findings of previous studies (Rabey et al. 2015; Lieber and Fridén 2000; Wilson et al. 2004). The best model of this relationship includes only the specific hindlimb muscle (AICc = 103.23; ANOVA: *F*_(3)_ **=** 8.55, *p* **<** 0.001) as the explanatory variable (Supporting Information S2: Table S3). The parallel‐fibred muscles have a significantly smaller PCSA than both the pennate muscles, while there are no significant differences within each muscle type (Table 1 and Figure 4). However, it is worth noting that both terrestrial jumpers and walker‐hoppers have significantly higher PCSA values than swimmers for the cruralis in the phylogenetic version of this analysis (Supporting Information S2: Table S5).

**Table 1:** Tukey HSD results from the best ANOVA model for size‐corrected physiological cross‐sectional area. Meeting the *p* < 0.05 significance threshold.

Pairwise comparison	Difference in means	*p* value
Muscle type		
Cruralis—Gluteus magnus	1.468	**0.001**
Cruralis—Semimembranosus	0.971	**0.048**
Cruralis—Plantaris longus	0.025	1
Plantaris longus—Gluteus magnus	1.493	**0.001**
Plantaris longus— Semimembranosus	0.996	**0.040**
Gluteus magnus— Semimembranosus	0.497	0.514

**Figure 5:**
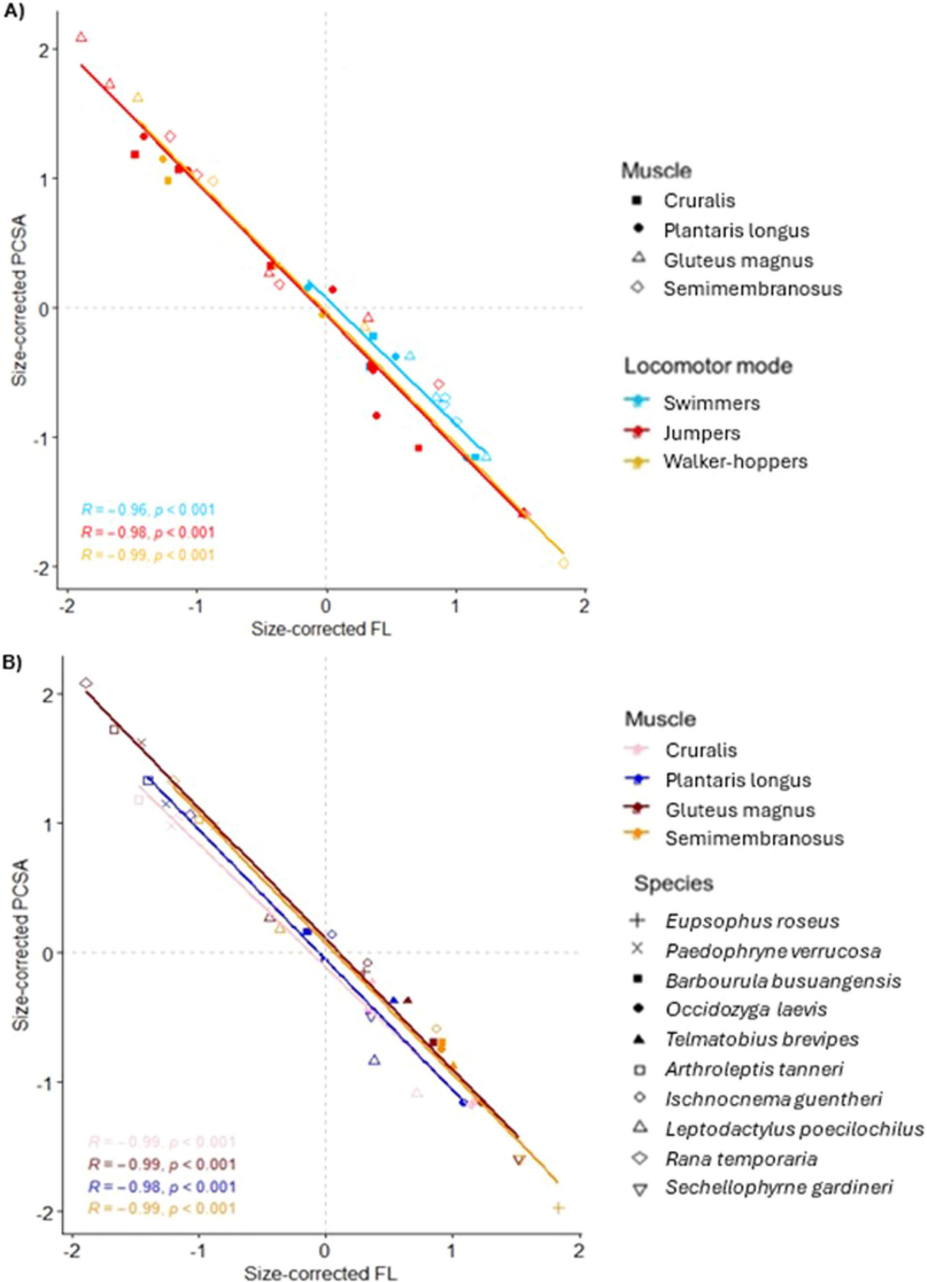
The relationship between size‐corrected fibre length (FL) and size‐corrected physiological cross‐sectional area (PCSA) when all muscles have a volume equal to 1mm^3^. There are four points per species; one for each muscle. Data are the same across the two plots, with points colour‐coded according to either (A) locomotor mode or (B) muscle type. In (A), filled shapes represent pennate muscles, and empty shapes represent parallel‐fibred muscles. In (B), empty shapes represent jumpers, filled shapes represent swimmers, and cross‐type shapes represent walker‐hoppers. PCSA for the parallel‐fibred muscles simply represents the anatomical CSA (i.e., pennation angle = 0). The grey dashed lines represent the means across each axis, which divide the plot into the four areas of functional space. The statistics reported refer to Pearson's correlation test.

To account for variation in PCSA simply due to differences in muscle volume, rather than fibre architecture alone, an additional ANOVA was performed where PCSA had been calculated with a fixed muscle volume of 1 mm^3^ for all species (Figure 5). The best model contained only locomotor mode as the explanatory variable (AICc = 114.97; ANOVA: *F*_(2)_ = 4.099, *p* = 0.025). Specifically, jumpers have a significantly higher PCSA than swimmers (Tukey HSD: difference = 0.897, *p* = 0.035) when muscle volumes are equal. This is largely because jumpers have significantly shorter fibres, as there are no significant differences in pennation angle between locomotor modes for both the plantaris longus (Kruskal–Wallis: Chi‐squared = 2.022, *p* = 0.364) and cruralis (ANOVA: *F*_(2)_ = 0.330, *p* = 0.73).

## 4 Discussion

Despite fibre architecture being an important determinant of muscle function, it is understudied across a wide range of ataxa, largely due to a lack of technology capable of accurately extracting and measuring lots of fibres without destroying the specimen (Charles et al. 2022). Here, we present the first digital fibre analysis of frogs in relation to their locomotor mode. We find that the trade‐off between size‐corrected PCSA and fibre length, and thus muscle force output, contractile speed and range of motion, can differ significantly between jumpers and swimmers, but not walker‐hoppers (partially supporting H1). Where species place on this functional spectrum of fibre architecture does largely depend on the muscle being examined (supporting H2), primarily due to differences in fibre length, rather than pennation angle. Overall, this study presents novel insights into how frogs utilise fibre architecture to address the requirements of different locomotor modes and discusses several promising directions for future comparative analyses.

### 4.1 Contrasting Modifications to Fibre Architecture Among Locomotor Modes

Frogs specialising in different locomotor modes may adapt their fibre architecture to perform functions effectively in different ways. The results presented here support the conclusions of previous studies, where jumping is a strong driver of changes in frog musculoskeletal physiology (Nauwelaerts, Ramsay, and Aerts 2007; Leavey, Richards, and Porro 2024). Jumpers have a significantly higher PCSA across muscles than swimmers only when all muscles are scaled to the same volume (Figure 5). This suggests that, for a given volume, the fibre architecture of jumpers is specialised to increase the force‐generating capacity of their muscles (Mendoza and Azizi 2021), while swimmers ‘spend’ their volume in a way that prioritises fibre length over force. This supports previous studies of function, where smaller muscle forces and lower rates of muscle shortening were found during swimming compared to jumping, even within the same frog (Calow and Alexander 1973). This difference between PCSAs is mainly driven by the cruralis (Supporting Information S2: Table S5), which has been shown to negatively affect swimming performance when it has a larger cross‐sectional area (Nauwelaerts, Ramsay, and Aerts 2007). Swimmers have a comparatively narrow range of fibre lengths (Figure 4) and are the only group to show a significant increase in fibre length with muscle length (Figure 3). These results suggest that the fibre lengths in swimmers are under stronger selection than other locomotor modes, or that their range of fibre lengths are constrained by some other factor. Swimmers may invest more strongly into longer fibres because they require larger ranges of motion, since swimming is used to navigate their environment, while jumping is a one‐off, consistent movement. Alternatively, swimmers could afford to rely primarily on increases in muscle mass to increase force ouput, as spending the majority of their life in a buoyant medium could reduce the considerable metabolic costs associated with transporting muscle mass.

### 4.2 Fibre Architecture May Be an Example of Many‐to‐One Mapping of Morphology to Function

There are fewer significant differences in fibre architecture between locomotor modes than expected. For example, unlike jumping, walking performance has been shown to be uncorrelated with the contractile properties of the semimembranosus and plantaris longus, and is strongly associated with small hindlimb mass (Astley 2016). However, walker‐hoppers were not significantly different from jumpers in any of our analyses. Our results could therefore indicate that there is high plasticity in muscle use across species (Vera et al. 2022). Using two jumpers as an example, *Sechellophryne gardineri* has muscles in the force, power, and displacement areas of functional specialisation (Figure 4) due to the high variation in fibre length across its muscles. In comparison, *Arthroleptis tanneri* has a small range of short fibres, with the only significant variation between the PCSA of each muscle being due to volume. *S. gardineri* is therefore likely to have a greater range of contractile velocities, as well as versatility in the functional workspace of the hindlimb (Lieber and Fridén 2000). Since this variation in fibre architecture occurs even when the locomotor mode is the same, frogs could be using different fibre anatomies to achieve similar functions, thus alleviating any selection pressures to alter larger components of muscle or bone structure. Fibre architecture could therefore be an example of many‐to‐one mapping (Moen 2019; Wainwright 2005). Direct functional studies are needed to test that this is not just a case of differences in jumping ability (see Section 4.6).

The lack of significant differences for the walker‐hopper group could simply reflect its small sample size. For example, the muscles of *Paedophryne verrucosa* all place highly in the force specialist region of the functional plot (Figure 5) despite its poor jumping abilities (Rittmeyer et al. 2012). A supplementary analysis showed that the only difference that removing walker‐hoppers from our data set made was that the differences in PCSA between the semimembranosus and both of the pennate muscles are no longer significant (Supporting Information S2: Table S7). This is likely due to similarities in volume across these muscles in jumpers and swimmers, since there are still significant differences in fibre length (Supporting Information). There is likely to be other determinants of muscle architecture which could be driving this high variability in fibre architecture between locomotor modes, such as phylogenetic history, habitat type, location within a dispersal range (Padilla, Courant, and Herrel 2019), and body size (Böhmer et al. 2018; Bishop, Wright, and Pierce 2021) (see Section 4.6).

### 4.3 Where Species Place in Functional Space Depends on the Muscle Being Examined

Long fibres enable parallel‐fibred muscles greater range and control of hindlimb motion, while large pennate muscles with short fibres are important for generating explosive movement due to the dense packing of muscle fibres (Enriquez‐Urzelai et al. 2015; Calow and Alexander 1973; Bishop, Wright, and Pierce 2021). These functional trends are reflected in the fibre architecture of most of our study taxa, irrespective of locomotor mode. The contractile speed specialist area of the functional space plot is occupied primarily by parallel‐fibred muscles (Figure 4). The exceptions were the cruralis and plantaris longus of *Telmatobius brevipes* (swimmer) and the plantaris longus of *Ischnocnema guentheri* (jumper) (Figure 4). The remainder of the pennate muscles all occupy the force or power specialist regions. The only parallel‐fibred muscle in the power region was the semimembranosus of *Occidozyga laevis* (swimmer). The semimembranosus from two jumpers—*I. guentheri* and *A. tanneri* (jumpers)—sits clearly within the force specialist region of the plot, while the gluteus magnus of *I. guentheri* and the semimembranosus of *P. verrucosa* sit just on the boundary between force and ‘economy’ specialist. The ‘economy’ region is only ever occupied by parallel‐fibred muscles. This demonstrates that locomotor mode can sometimes influence muscles to sit in regions outside of the usual specialism. There appears to be an upper limit for muscle power defined by muscle mass, since only the plantaris longus of *I. guentheri* occupies the power specialist region when volumes are equal (Figure 5). Essentially, no level of fibre architecture optimisation can enable muscles to exert comparatively high power if the volume is low. This is likely because muscles in this specialist region experience considerably higher metabolic costs (Martin et al. 2020). Functional studies will be needed to check whether the trends observed here reflect *in vivo* locomotor performance, for example, whether the better jumpers within the jumper group have more highly adapted fibre architecture.

### 4.4 Fibre Architecture That Enables Higher Power Output Does Not Necessarily Reduce the Need to Grow and Maintain High Muscle Mass

Astley (2016) suggested that the evolutionary lability of muscle contractile properties could allow organisms to increase muscle power output without increasing muscle mass, thus reducing the large metabolic costs associated with greater muscle size. In terms of fibre architecture, Mendoza and Azizi (2021) observed three frog species and found that increases in plantaris longus pennation angle explained the differences in mass‐specific forces, and therefore jumping ability (likely via increased storage and recoil of tendon elastic energy). Though we note that increased PCSA does not necessarily confer increased muscle power in the absence of a tendon. By analysing PCSA calculated from equal muscle volumes, we found that while fibre length is a considerable driver of differences between locomotor modes, there are no significant differences in the pennation angle, and the power‐specialist region of the functional space plot remained largely unoccupied (Figure 5). Additionally, we found no significant relationships between muscle mass, fibre length (Supporting Information S2: Figure S5) and pennation angle (Supporting Information S2: Figure S6 and Table S6) in this supplementary analysis. Since power output is known to vary among frogs independently of size (Roberts, Abbott, and Azizi 2011), we can only speculate cautiously that these results imply a lack of evidence that fibre architecture might allow frogs to increase muscle power output without increasing muscle mass. These results also imply that pennate muscles may not be built with the purpose of maximising potential force output (i.e., shorter fibres and higher pennation angles with increasing muscle mass). If we hypothetically assume muscles have similar mass‐specific power, our findings suggest that smaller volume muscles could not match or exceed the power output of larger muscles (Figures 4 and 5). This is not surprising, given that optimising fibre length and pennation angle alone (i.e., geometry) is not sufficient for increasing intrinsic muscle power; an increase in volume (mass) would be necessary. Future studies directly measuring the muscle (or whole animal) forces for these species will be needed to fully address this. However, we do find that the gluteus magnus shows a significant increase in fibre lengths with muscle *length* irrespective of body size (Figure 3), indicating that it might experience stronger selection pressures to increase potential contractile speed and range of knee extension, or could be under stronger developmental constraints. Future investigation using appropriate mechanical testing is required to rule out developmental constraints versus selection for functional reasons, and to explore the potential trade‐offs present between muscle size and architecture.

### 4.5 Limitations

Fibre tracking is only as successful as the quality of the contrast‐enhanced μCT scan. The primary limitation of our study is sample size, which is limited by scan resolution to 10 species across three locomotor groups, and to four muscles in one individual per species, thus missing an important component of within‐species variability. However, it is worth noting that this is a substantial dataset for a diceCT study. Architectural variables from previous studies would have ideally been included in our analyses to expand the range of taxa, but the mean fibre lengths obtained here appear smaller than those recorded in studies whose method for body size correction (or lack thereof) allowed for more direct comparisons (Astley 2016; Kargo and Rome 2002). This difference could potentially be because we use formalin‐fixed and iodine‐stained specimens, which are known to suffer from tissue shrinkage (Böhmer et al. 2018), while previous studies used fresh specimens. However, differences in the level of shrinkage experienced by the individual fibres due to differences in preservation and staining procedures would be proportional to that of the entire muscle. Additionally, the *GoodFibes* package has undergone substantial testing to ensure that comparable results are obtained between the traditional and digitally automated methods for fibre extraction (Arbour 2023), and there are no non‐phylogenetic models where the addition of grayscale cut‐off improved model fit (Supporting Information S2: Tables S2 and S3). Most studies also do not disclose exactly how many fibre measurements they took. Compared to those that did (Nauwelaerts, Ramsay, and Aerts 2007; Lieber and Brown 1992), we measured considerably more fibres, making PCSA estimations less prone to error (Charles et al. 2022). To conclude, while this study provides novel insights into the relative differences in architectural variables between frogs specialising in different locomotor functions, future comparisons made using the raw data should be taken with caution.

A recent review study has stated that measuring pennation angle in just a few areas within a static muscle can lack functional significance (Lieber 2022). This is largely because muscle fibres can differ in orientation throughout the muscle, particularly when comparing deep and superficial regions (Charles et al. 2022; Azizi and Deslauriers 2014), and will rotate during contraction such that the shortening of the fibres is smaller than the total shortening of the muscle (Roberts et al. 2019). Pennate muscles experience variable gearing throughout movement (i.e., fibres act differently depending on the mechanical load), which can affect how muscle moment arms change with changes in joint angle, as well as the size of a muscle's functional range (Azizi and Roberts 2014; Azizi, Brainerd, and Roberts 2008). This creates regional variation in mechanical output both throughout the muscle and throughout any movement (Azizi and Deslauriers 2014). Additionally, any pennation angle below 30°, which is common for most pennate frog muscles (Kargo and Rome 2002; Supporting Information S1: Dataset), is likely to have little effect on force calculations, as the cosine variable is then typically close to one (Equation 1; Böhmer et al. 2018). Therefore, since extensive fibre extractions and measurements is a very time‐consuming process, many studies have treated pennation angle as constant throughout the muscle (Calow and Alexander 1973; Astley 2016; Mendoza and Azizi 2021; Kargo and Rome 2002; the present study), or all muscles are treated as parallel‐fibred (Nauwelaerts, Ramsay, and Aerts 2007). Ultimately, examining fibre architecture in isolation may not relate to muscle function in the same way once placed in the skeletal system. More detailed sensitivity tests are needed to estimate how much this variation in fibre orientation directly affects force production (see Section 4.6).

### 4.6 Future Directions

Given the primary limitation of the present study being sample size, improving scan resolution and staining techniques will be crucial for future studies comparing fibre architecture across vertebrates. Improvements in the ability to visualise fibre structure will be vital for extracting an accurate phylogenetic signal to examine how muscle architecture and function changes throughout evolutionary history. Broadening the sample across a wider range of body sizes would also facilitate a more comprehensive investigation into the potential impact of allometry, on fibre architecture and fibre number, especially in miniaturised vertebrates (Supporting Information S2: Figure S4). Recent evidence shows that small frogs may benefit more from tendon elastic recoil than larger frogs (Sutton et al. 2019); thus, small frogs may rely more strongly on tendons to increase power output to compensate for limitations on body size. Ideally, arboreal jumpers and burrowers would have also been included in the data set because, as with gross muscle structure, fibre architecture could be a functional mediator between habitats in frogs (Leavey, Richards, and Porro 2024). For instance, the forelimb muscles in arboreal pine martens (Böhmer et al. 2018) and hindlimb muscles in arboreal squirrels (Nyakatura et al. 2019) have been shown to have greater force‐producing capabilities compared to their close terrestrial relatives due to differences in fibre architecture. Additionally, several muscles were unable to be included in this study as their resolution was not clear enough for fibre tracking across all species. It would have been ideal to investigate whether the adductors (important during jumping, for example, adductor magnus) and abductors (important during swimming, e.g., iliacus externus) are a key point of difference in fibre anatomy between these locomotor modes (Nauwelaerts, Ramsay, and Aerts 2007). It is also unknown how the separation of muscle into distinct heads (Leavey, Richards, and Porro 2024) impacts fibre architecture, and therefore the trade‐off between muscle force and contractile speed. Furthermore, future studies should investigate how the presence/absence of the tendinous insertion in the semimembranosus and gracilis major may impact function during thigh movement (Leavey, Richards, and Porro 2024). Finally, it would be interesting to see whether the tibialis anticus longus and extensor cruris brevis, which are small pennate muscles in the shank (Collings and Richards 2019), differ in their fibre architecture between locomotor modes in the same ways as they significantly differ in size (Leavey, Richards, and Porro 2024). Previous studies have observed muscles becoming increasingly pennate when moving distally down the limb across other vertebrates (Powell et al. 1984; Charles et al. 2016), which is said to improve energy conservation during locomotion (Sacks and Roy 1982). Having smaller distal segments is important for reducing moments of inertia (i.e., the forces required for limb acceleration; Nauwelaerts, Ramsay, and Aerts 2007), so the shank may be more limited in how much it can increase force output through increases in muscle mass. The muscles in the shank may therefore be under stronger selection pressures to optimise fibre architecture than those in the thigh.

A potential solution for addressing the issue of fibre resolution could be to induce muscle shrinkage by prolonged dehydration and/or higher iodine concentrations during staining, as this has been observed to result in better separation of individual fibres (Dr. Edward Stanley, personal communications). However, facilitating easier fibre detection in this way would have consequences for accurately measuring muscle mass, volume and length. Future studies could attempt staining for gross measurements first, then increasing the concentration of the staining solution so that fibres can be better examined in subsequent µCT scans. For subsequent statistical analyses, the appropriate size correction would then be included to account for the additional muscle shrinkage.

Computational models may be key to quantifying the impact of variation in musculoskeletal anatomy on function. Previous studies have used fibre architecture to better inform and validate biomechanical models (Sanchez et al. 2013; Sellers et al. 2017; Orsbon et al. 2020), which is particularly important for estimating locomotor performance in extinct taxa (Bishop et al. 2021), but this has not been done in frogs before. Muscle volume, fibre length and pennation angle can be systematically varied while keeping all other features of anatomy the same to examine how these model parameters impact motion. Additionally, variation in the pennation angle throughout the layers of an individual muscle, and its effect on mechanical output, could be explored through a series of sensitivity tests without the need for extensive dissection (Azizi, Brainerd, and Roberts 2008). In turn, a computational approach will facilitate future advances in ‘palaeomyology’ (Perry and Prufrock 2018), where researchers can virtually graft different potential fibre anatomies onto fossilised remains and test how they impact function. If sufficient scan resolution is acquired, the muscle fibres in *Ascaphus*, the most basal living frog which evolved over 160 million years ago, would be an interesting variable to include in musculoskeletal models of locomotor function in extinct taxa using the methods from Sánchez et al (2014). Ultimately, musculoskeletal models would provide more direct evidence of how differences in hindlimb muscle architecture affect hindlimb multifunctionality, and therefore versatility in locomotor function.

## 5 Conclusion

By examining a combination of musculoskeletal variables (muscle mass, fibre length, physiological cross‐sectional area, skeletal structure) using digital techniques, we can derive the best possible estimates of muscle function during locomotion, and therefore their ecological relevance. This paper complements the comparative anatomical investigation of gross muscle structure of Leavey, Richards, and Porro (2024) by exploring how the functional spectrum of architectural properties in frog muscle fibres differs between four hindlimb muscles with varying roles in locomotion. Jumpers show specialisation towards producing higher forces than other locomotor modes. The high plasticity in fibre architecture observed within each locomotor group indicates that many‐to‐one mapping of fibre form to function is occurring, and that there are other determinants of fibre architecture besides locomotor mode. We also exemplify how digital techniques can enable the quantification of muscle architecture in some of the world's smallest vertebrates, providing the foundation for future studies to determine the effects of miniaturisation on anatomy and function. Ways in which the acquisition of this anatomical information can be improved, and how it can be incorporated into musculoskeletal dynamics models, have also been suggested.

## Author Contributions

**Alice Leavey:** conceptualisation, investigation, writing–original draft, writing–review and editing, formal analysis, data curation, methodology, validation, visualisation, project administration, resources. **Christopher T. Richards:** writing–review and editing, supervision, funding acquisition, project administration. **Laura B. Porro:** writing–review and editing, supervision, funding acquisition, project administration.

## Conflicts of Interest

The authors declare no conflicts of interest.

### Peer Review

The peer review history for this article is available at https://www.webofscience.com/api/gateway/wos/peer-review/10.1002/jmor.70016.

## Supporting information

Supporting information.

Supporting information.

## Data Availability

The data that supports the findings of this study are available in the supplementary material of this article.
