# Supplementary material for: Frog Fibres: What Muscle Architecture Can Tell Us About Anuran Locomotor Function
Source: J Morphol. 2024 Dec 17;286(1):e70016. doi: 10.1002/jmor.70016 (PMC11652814; doi:10.1002/jmor.70016)
Supplement: Supplementary file 2 — Supporting information. [file JMOR-286-e70016-s001.docx]

**Supplementary information**

##### Preparing image stacks

Muscle image stacks were imported into ImageJ prior to fibre tracking in R to increase the contrast of the fibres using the ‘unsharp mask’ filter (Figure S1). This sharpening operator enhances the edges in an image by subtracting a smoothed version of the image from the original image, and ultimately increases the contrast between the muscle fibres and interstitial spaces (Dr Jaimi Gray, pers. comms). The mask weight (i.e., the strength of the filtering) was set to 0.9.


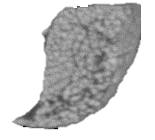

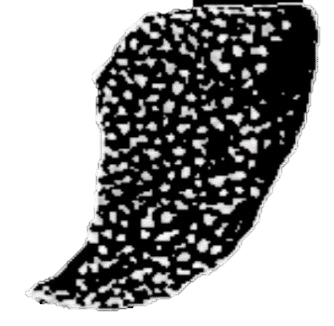

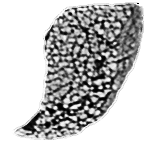


‘Unsharp mask’ = 0.9

ImageJ

Cut-off = 0.6

R - *GoodFibes*

Figure S1 - The process of muscle image stack preparation

, using the gluteus magnus muscle from *Eupsophus roseus* (voucher number YPM:HERR:005002) as an example. The first step involves the use of a filter to increase contrast in ImageJ, while the second step defines the grayscale values below which voxels will be considered black by the fibre tracking algorithm utilised by the ‘good.fibes’ function in R.

##### Measuring pennation angle

Figure S2 illustrates how pennation angle for the cruralis and plantaris longus was measured from each µCT scan.


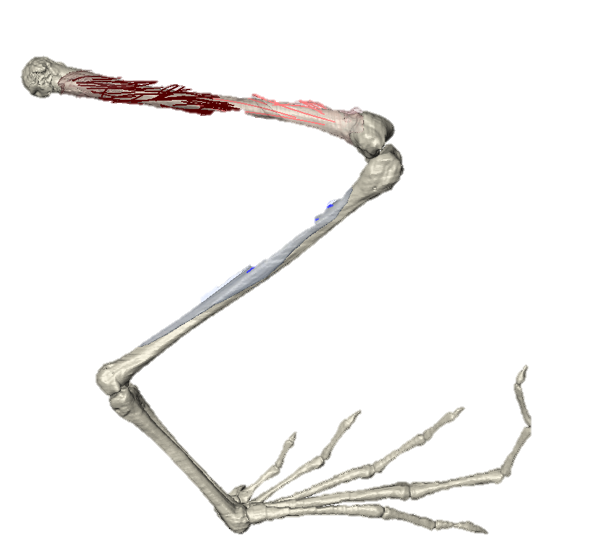

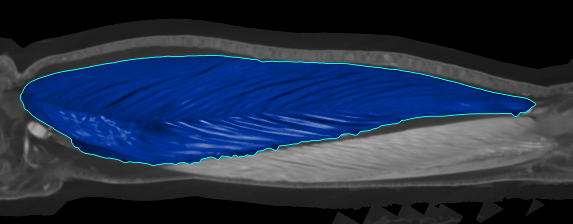

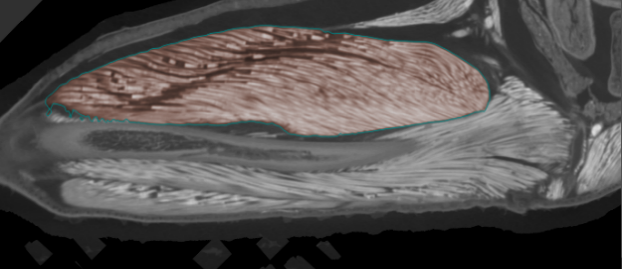


Figure S2 - Measurement of pennation angle along the force-producing axis of the cruralis and plantaris longus, using *Arthroleptis tanneri* (voucher number CAS:HERP:168823)

in VGStudio Max (Version 3.4) as an example. The red lines on the 3D model (left) represents where the views in the 2D plane are from (right).

Cruralis

Plantaris longus


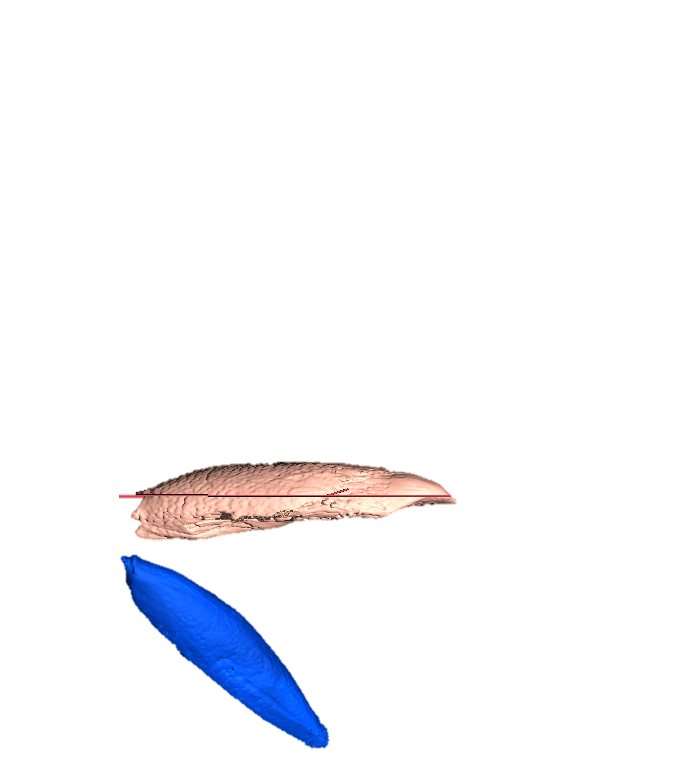


##### Testing for allometry

##### As many of the variables describing muscle architecture are highly correlated with measures of body size (Table S1), we chose to size-correct our data prior to further analyses.

Table S1 - The test statistics and *p*-values for Spearman’s rank correlation tests between log-transformed body size variables (snout-vent length and total estimated body mass) and log-transformed muscle architecture variables (fibre length (FL), physiological cross sectional area (PCSA), and muscle belly volume (MBV) and length (MBL)).

| **Model** | rho | *p* |
| --- | --- | --- |
| FL ~ SVL | 0.930 | **< 0.001** |
| PCSA ~ body mass | **0.741** | **< 0.001** |
| MBV ~ body mass | 0.800 | **< 0.001** |
| MBL ~ SVL | 0.903 | **< 0.001** |

##### Normality tests

##### After body size correction, the resulting residuals were checked for normal distribution while controlling for phylogenetic relatedness using the Portik *et al.* (2023) tree before subsequent statistical analyses (Table S2).

Table S2 - The test statistics and *p*-values for Shapiro-Wilk normality tests for pennation angle (PA), size-corrected fibre length (FL), size-corrected physiological cross sectional area (PCSA), and size-corrected muscle belly mass (MBM) and length (MBL). Data which are not normally distributed have been highlighted in bold.

|  | **Semimembranosus** | | **Gluteus magnus** | | **Cruralis** | | **Plantaris longus** | |
| --- | --- | --- | --- | --- | --- | --- | --- | --- |
|  | L | *p* | L | *p* | L | *p* | L | *p* |
| PA | NA | NA | NA | NA | 0.911 | 0.293 | **0.836** | **0.039** |
| FL | 0.930 | 0.447 | 0.983 | 0.980 | 0.956 | 0.737 | 0.915 | 0.314 |
| PCSA | **0.844** | **0.049** | 0.848 | 0.055 | 0.892 | 0.177 | 0.868 | 0.094 |
| MBM | 0.972 | 0.910 | 0.956 | 0.742 | 0.959 | 0.778 | 0.903 | 0.234 |
| MBL | 0.857 | 0.070 | 0.964 | 0.830 | **0.840** | **0.044** | 0.900 | 0.221 |

##### Phylogenetic analyses of fibre architecture

Since our species sample size is small due to the constraints of Cfibre resolution, accurate estimates of the influence of shared phylogenetic history cannot be obtained (Münkemüller *et al.,* 2012). Indeed, all but one evolutionary model for size-corrected fibre length and size-corrected physiological cross-sectional area (PCSA) was the ‘white noise’ model, i.e., no phylogenetic signal. The only exception was the semimembranosus PCSA, which had Brownian motion as the best evolutionary model (log-likelihood = -10.52, AIC = 25.04, weight = 0.377). While it showed some phylogenetic signal, it was not significant (W = 0.843, *p* = 0.132). Hence, the main analyses presented in this manuscript do not test for phylogenetic relatedness. Where possible we ran a supplementary set of phylogenetic analyses using the Brownion Motion model of evolution to address each hypothesis to double check that there were not any differences compared to our main set of results. Where species from our dataset are not present in the Portik *et al.* (2023) phylogeny (*Paedophryne verrucosa, Telmatobius brevipes,* and *Occidozyga laevis*), we used the most closely related congeneric taxa for estimations of branch lengths:

- *Paedophryne oyatabu –* the only species from this genus in the Portik *et al.* tree.
- *Telmatobius macrostomus -* Gómez, R.O., Ventura, T., Turazzini, G.F., Marivaux, L., Flores, R.A., Boscaini, A., Fernández‐Monescillo, M., Quispe, B.M., Prámparo, M.B., Fauquette, S. and Martin, C., 2024. A new early water frog (*Telmatobius*) from the Miocene of the Bolivian Altiplano. *Papers in Palaeontology, 10(1),* p.e1543.
- *Occidozyga berbeza* - Chen, W.C., Peng, W.X., Liu, Y.J., Huang, Z., Liao, X.W. and Mo, Y.M., 2022. A new species of *Occidozyga* Kuhl and van Hasselt, 1822 (Anura: Dicroglossidae) from Southern Guangxi, China. *Zoological Research*, *43*(1), p.85.


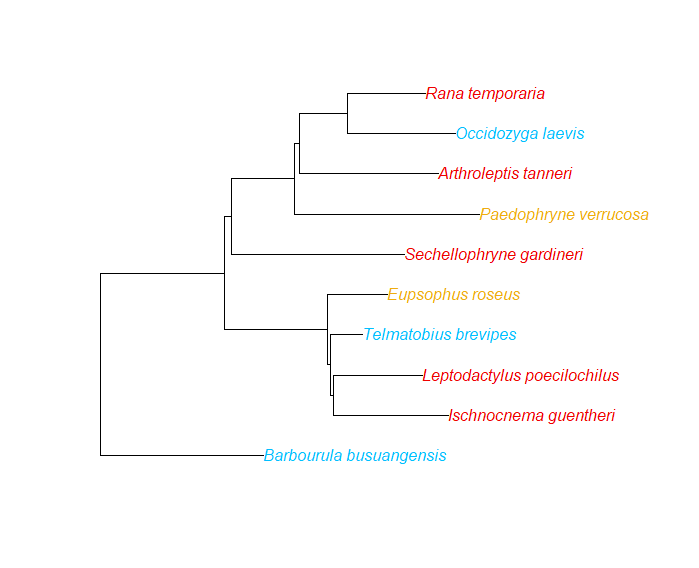


Figure S3 – The phylogenetic relationships between our study taxa using the Portik *et al.* (2023) tree, coloured according to their locomotor mode (red = terrestrial jumper; blue = swimmer; yellow = walker-hopper).

The best phylogenetic models of fibre length (Table S3) and PCSA (Table S4) use only locomotor mode as the explanatory factor - adding the grayscale cut-off does not improve the fit of the model, thus assuring there is no bias in fibre extraction affecting the results. There were no significant differences between locomotor modes in fibre length for any of the four muscles. There was, however, one significant difference between locomotor modes in PCSA – the cruralis has a larger PCSA in jumpers and walkers than swimmers (Table S5).

Table S3 - The phylogenetic ANOVA models testing for differences between locomotor modes (LM) for size-corrected fibre length. Corrected Akaike’s Information Criterion (AICc) were used since sample size was small. The best model was always without the grayscale cut-off, but none of the best models showed significance values above *p* = 0.05 besides the intercept.

| **Muscle** | **Model** | **Log-likelihood** | **AICc** | **AICc weights** |
| --- | --- | --- | --- | --- |
| Plantaris longus | Cut-off + LM | -10.056 | 28.969 | 0.329 |
|  | LM | -10.104 | 27.542 | 0.671 |
| Cruralis | Cut-off + LM | -10.926 | 30.709 | 0.346 |
|  | LM | -11.049 | 29.431 | 0.654 |
| Semimembranosus | Cut-off + LM | -13.363 | 35.583 | 0.358 |
|  | LM | -13.54 | 34.414 | 0.642 |
| Gluteus magnus | Cut-off + LM | -14.057 | 36.972 | 0.385 |
|  | LM | -14.351 | 36.035 | 0.615 |

Table S4 - The phylogenetic ANOVA models testing for differences between locomotor modes (LM) for size-corrected physiological cross-sectional area (PCSA). Corrected Akaike’s Information Criterion (AICc) were used since sample size was small. The best model was without the grayscale cut-off for all muscles except the gluteus magnus.

| **Muscle** | **Model** | **Log-likelihood** | **AICc** | **AICc weights** |
| --- | --- | --- | --- | --- |
| Plantaris longus | Cut-off + LM | -11.97 | 32.797 | 0.327 |
|  | LM | -12.011 | 31.355 | 0.673 |
| Cruralis | Cut-off + LM | -9.685 | 28.228 | 0.486 |
|  | LM | -10.392 | 28.117 | 0.514 |
| Semimembranosus | Cut-off + LM | -9.533 | 27.923 | 0.33 |
|  | LM | -9.568 | 26.47 | 0.67 |
| Gluteus magnus | Cut-off + LM | -7.759 | 24.374 | 0.915 |
|  | LM | -10.9 | 29.133 | 0.085 |

Table S5 - The pairwise results of the phylogenetic ANOVA model testing for differences between locomotor modes (LM) for size-corrected physiological cross-sectional area (PCSA) of the cruralis. No other muscles tested showed significant differences between locomotor modes. SE = standard error and *p*-values above the 0.05 significance threshold have been highlighted in bold.

| **Coefficient** | **Estimate** | **SE** | **t-value** | ***p*-value** |
| --- | --- | --- | --- | --- |
| Intercept | -0.057 | 0.587 | -0.097 | 0.926 |
| Jumper | 1.119 | 0.433 | 2.584 | **0.036** |
| Walker | 1.449 | 0.524 | 2.765 | **0.028** |

Table S6 - The result of the phylogenetic least squares (PGLS) model testing for significant relationships between size-corrected muscle belly mass and size-corrected fibre length (FL) and, for pennate muscles, the pennation angle. SE = standard error.

| **Muscle** | **Model** | **Estimate** | **SE** | **t** | **p** |
| --- | --- | --- | --- | --- | --- |
| Semimembranosus | Intercept | -0.409 | 0.570 | -0.718 | 0.493 |
|  | FL | 0.341 | 0.214 | 1.589 | 0.151 |
| Gluteus magnus | Intercept | -0.681 | 0.788 | -0.864 | 0.413 |
|  | FL | 0.228 | 0.275 | 0.830 | 0.431 |
| Cruralis | Intercept | 0.494 | 0.837 | 0.590 | 0.571 |
|  | FL | 0.170 | 0.403 | 0.420 | 0.685 |
|  | Intercept | -0.046 | 2.278 | -0.020 | 0.984 |
|  | Pennation | 0.021 | 0.081 | 0.256 | 0.805 |
| Plantaris longus | Intercept | 0.560 | 0.832 | 0.643 | 0.520 |
|  | FL | 0.382 | 0.433 | 0.883 | 0.403 |
|  | Intercept | -3.198 | 2.623 | 1.219 | 0.258 |
|  | Pennation | 0.175 | 0.118 | 1.479 | 0.177 |

**Number of muscle fibres**

Figure S4 demonstrates how different frogs can have different numbers of fibres in their muscles even if part of the same locomotor group, usually corresponding to differences in body size, rather than degree of pennation.

Figure S4 - A cross-section of the plantaris longus of the two walker-hopper species, exemplifying the difference in fibre number due to differences in body size. SVL = snout-vent length.


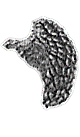

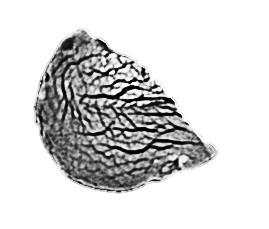


*Paedophryne verrucosa -* 8.36mm SVL, 22.71 degrees pennation angle

*Eupsophus roseus -* 35.1mm SVL, 19.06 degrees pennation angle

## The relationship between muscle size and architecture

Muscles could be specialised towards force production without the high metabolic costs associated with growing and maintaining large muscle size (i.e., shorter and more pennate fibres in smaller muscles), or pennate muscles may be built with the purpose of maximising potential force output (i.e., shorter and more pennate fibres in larger muscles) and parallel-fibred muscles could be adapted to maximise range of motion and contractile speed (i.e., longer fibres in larger muscles). Our supplementary set of analyses show that fibre length generally increases with increasing muscle mass for all hindlimb muscles and locomotor modes, but these relationships are not significant (Supplementary Figure S5). Pennation angle generally increases with muscle mass for plantaris longus, but not the cruralis, and for jumpers, but not walker-hoppers andswimmers, but again none of these relationships are significant (Figure S6). The supplementary phylogenetic generalised least squares test also shows a lack of significant results (Supplementary Table S6).


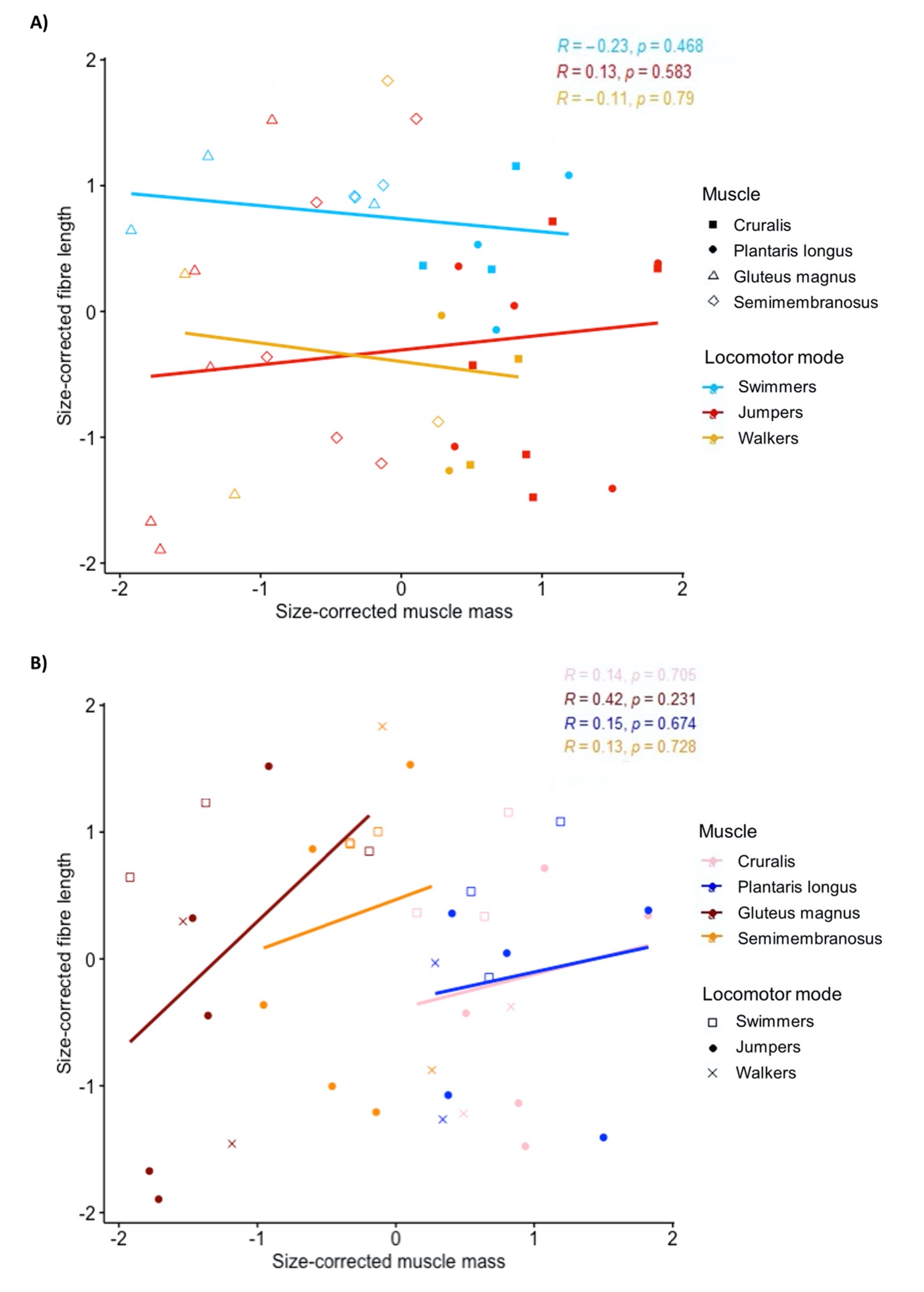

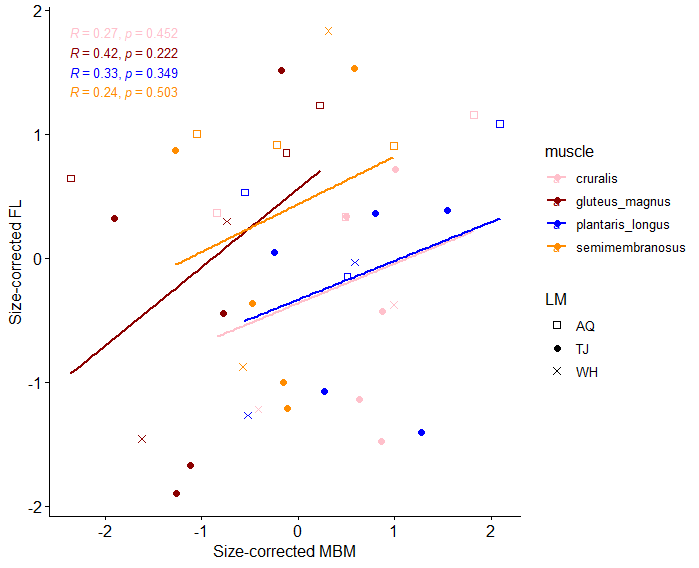

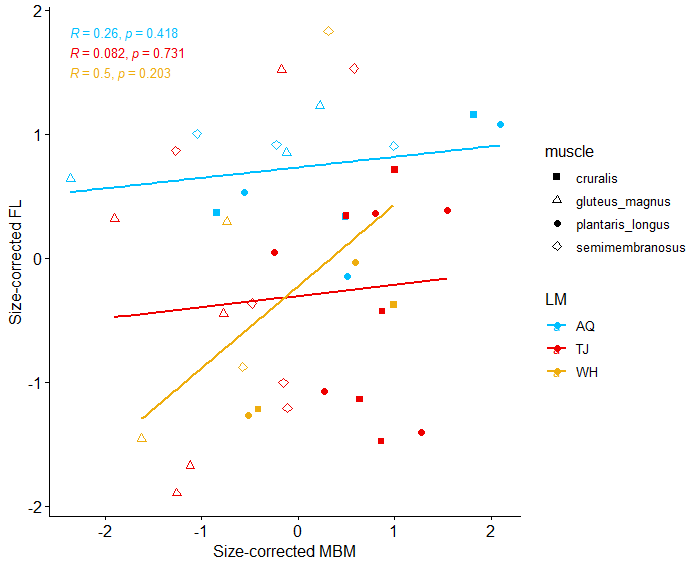


Figure S5 - The relationship between size-corrected fibre length and muscle mass

. There are four points per species; one for each muscle. The data are the same across the two plots, with points colour-coded according to either A) locomotor mode or B) muscle type. In A), filled shapes represent pennate muscles, and empty shapes represent parallel-fibred muscles. The statistics reported refer to Pearson’s correlation tests.

**Figure S6** - The relationship between average size-corrected muscle mass and pennation angle

B)


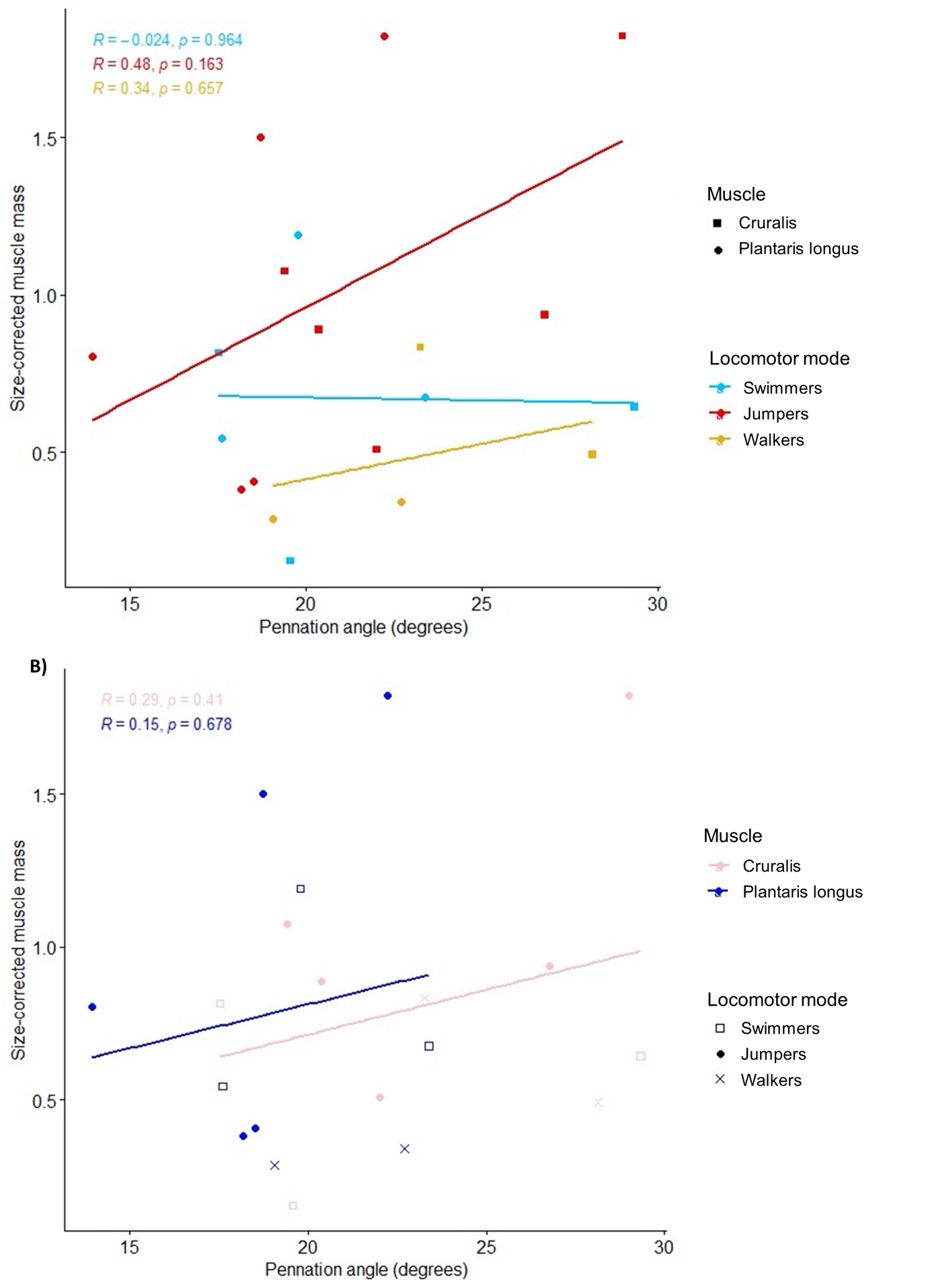

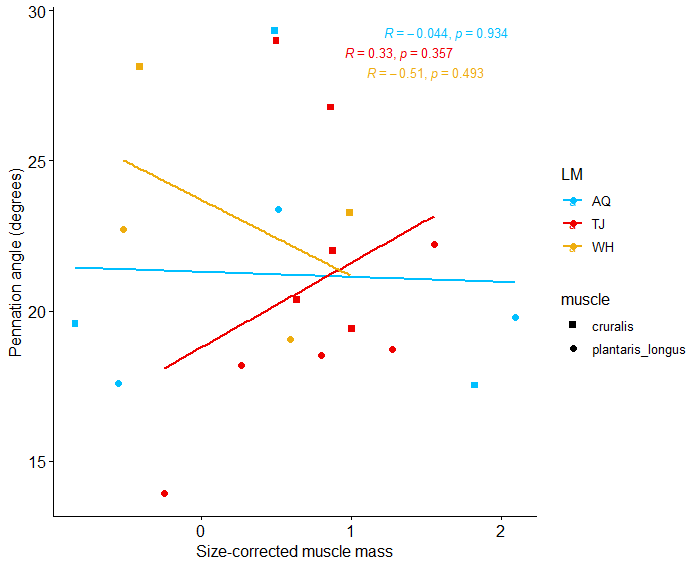

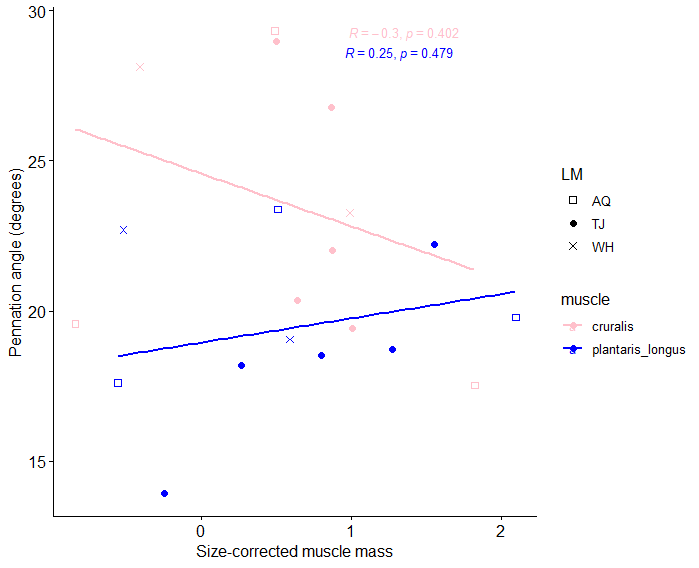


A)


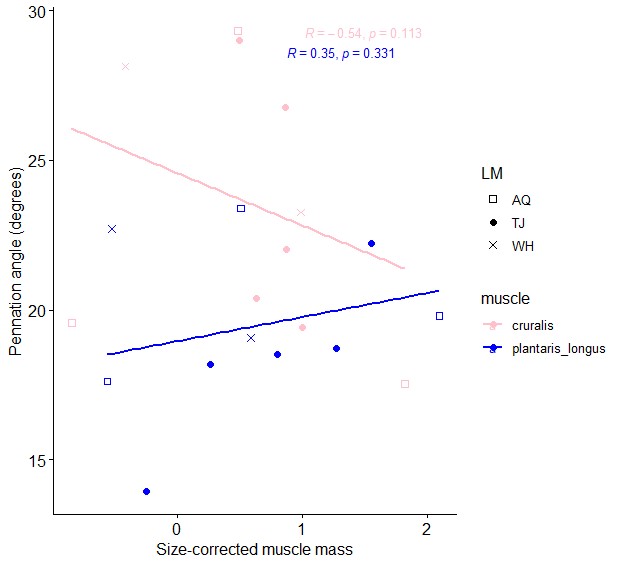


. There are four points per species; one for each muscle. The data are the same across the two plots, with points colour-coded according to either A) locomotor mode or B) muscle type. In A), filled shapes represent pennate muscles, and empty shapes represent parallel-fibred muscles. The statistics reported refer to Pearson’s correlation tests for all except the plantaris longus in B) which is a Spearman’s rank test.

**Supplementary tests without walker-hoppers**

We performed a supplementary version of our main analyses that excluded walker-hoppers from the dataset, since there are only two species. We found that the best model for fibre length was still the one which contained only locomotor mode as the explanatory variable (AICc = 86.23 compared to locomotor mode + muscle = 93.76). Again, we found that jumpers have significantly shorter fibres than swimmers (Tukey HSD: 1.041, *p* = 0.003). For PCSA, the model with both locomotor mode and muscle type (AICc = 90.93) narrowly bested the model containing only muscle type (90.97), but again there were no significant differences between jumpers and swimmers (Table S7). Without walkers in the dataset, the differences in PCSA between the semimembranosus and the cruralis, and the semimembranosus and plantaris longus are no longer significant (Table S7). When size-corrected PCSA is calculated with all muscle volumes are equal to one, the best model without the inclusion of walker-hoppers again contains only locomotor mode (AICc = 88.66), but this time the relationship is significant – jumpers have a significantly higher PCSA than swimmers (Tukey HSD: 0.897, *p* = 0.0103), largely due to differences in fibre length since there are no significant differences in pennation angle between jumpers and swimmers for both the cruralis (Tukey HSD: 1.372, *p* = 0.719) and plantaris longus (Dunn’s test: 0.745, *p* = 0.228).

Table S7 - Tukey HSD results from the best ANOVA model for size-corrected physiological cross-sectional area, excluding walker-hoppers from the dataset

. Results meeting the *p* < 0.05 significance threshold have been highlighted in bold.

| **Pairwise comparison** | **Difference in means** | ***p-*value** |
| --- | --- | --- |
| **Muscle type** | | |
| Cruralis – Gluteus magnus | 1.484 | **0.009** |
| Cruralis – Semimembranosus | 0.947 | 0.147 |
| Cruralis – Plantaris longus | 0.103 | 0.995 |
| Plantaris longus – Gluteus magnus | 1.587 | **0.005** |
| Plantaris longus – Semimembranosus | 1.050 | 0.092 |
| Gluteus magnus – Semimembranosus | 0.536 | 0.601 |
| **Locomotor mode** | | |
| Terrestrial jumpers - Swimmers | 0.518 | 0.11 |

# Supplementary Dataset

**Abbreviations used throughout the supplementary datasets:**

Locomotor mode:

- AJ - Arboreal Jumper
- TJ - Terrestrial Jumper
- WH – Walker-Hopper
- BWH – Burrower-Walker-Hopper
- AQ - Swimmer

Habitat type:

- Aquatic – spends the majority of its time in water.
- Arboreal – spends the majority of its time in vegetation/trees.
- Terrestrial – spends the majority of its time on the ground.
- Riparian – spends approximately an equal amount of time in water as it does in terrestrial environments, i.e., relies on water outside of just for the purposes of reproduction.

The raw data collected from this paper, where four muscles were analysed in ten species. This includes snout-vent length (SVL) and total body mass estimated from CT scan volume, the grayscale cut off used for each muscle for this function, the number of muscles fibres with high enough quality to been traced by the ‘good.fibes’ function in R, muscle belly volume (MBV), mean fibres lengths (FL) and their standard deviation, pennation angle, physiological cross-sectional area (PCSA), anatomical cross-sectional area (ASCA), and fibre length relative to muscle belly length (FL.MBL).
